# Precision in Facial Measurements: Comparative Analysis Between a Digital 3D Scanner and an Analog Instrument

**DOI:** 10.3390/dj13090395

**Published:** 2025-08-29

**Authors:** Francesco Puleio, Giorgio Lo Giudice, Angela Alibrandi, Ilenia Campione, Federica Di Spirito, Roberto Lo Giudice

**Affiliations:** 1 Department of Biomedical and Dental Sciences and Morphofunctional Imaging, Messina University, 98122 Messina, Italy; francesco.puleio@unime.it (F.P.); roberto.logiudice@unime.it (R.L.G.); 2 Unit of Statistical and Mathematical Sciences, Department of Economics, Messina University, 98122 Messina, Italy; angela.alibrandi@unime.it; 3 Department of Human Pathology of Adults and Developmental Age, Messina University, 98122 Messina, Italy; ilenia.campione@unime.it; 4 Department of Medicine, Surgery and Dentistry, University of Salerno, Via S. Allende, 84081 Baronissi, Italy; fdispirito@unisa.it

**Keywords:** facial scanner, 3D scan, surface imaging, dental scanner, photogrammetry, morphometrics, stereophotogrammetry

## Abstract

**Background:** Accurate facial proportion analysis is essential for therapeutic planning in dentistry. This study aimed to evaluate the Planmeca ProFace 3D scanner’s accuracy by comparing its digital measurements to analog caliper measurements. **Methods:** A comparative cross-sectional study included seven patients. Fourteen standardized facial landmarks were measured digitally and with an analog caliper. Distances were grouped as small (≤6.5 cm, Group A) or large (>6.5 cm, Group B). Paired *t*-tests, Cronbach’s Alpha, and Bland–Altman analysis assessed differences, reliability, and agreement. **Results:** The results showed a statistically significant difference between the two methods of measurements in group A (*p* = 0.016) and high statistical significance was obtained in group B (*p* = 0.001). Cronbach’s Alpha showed high reliability for Group A (α = 0.982) but low for Group B (α = 0.270). The mean difference between the caliper and software measurements was 0.24 ± 0.9 SD (min 0.16 max 2.92) in group A and 0.71 ± 2.8 SD (min 0.02 max 4.17). Bland–Altman analysis revealed a consistent positive proportional bias, with differences increasing for larger measurements. **Conclusions:** Facial point measurements by the means of digital scanning technique show measurements overlapping with analog technique for measurements less than or equal to 6.5 cm, with significant deviation for points with a distance greater than 6.5 cm. A hybrid approach or compensatory strategies are needed to ensure clinical precision.

## 1. Introduction

Analyzing and monitoring facial proportions, facial pathologies, and the growth of individuals with clefts, facial dysmorphisms, or anomalies in maxillary and mandibular bone growth, including malocclusions, is often essential to obtaining a correct and precise therapeutic plan. Interest in soft tissue evaluation and prediction of the post-intervention changes has been increasing in dentistry and maxillofacial-surgery fields as technology evolves [1]. Moreover, measuring facial proportions can offer valuable insights for designing proportionate and aesthetically pleasing prostheses [1,2,3].

The evolution of technology has seen a significant shift from conventional two-dimensional (2D) methods to advanced three-dimensional (3D) scanning and imaging techniques.

Yet, the gold-standard tool for linear distance measurements is currently the caliper. These measurements can be time-consuming and challenging, particularly when treating uncooperative patients. Furthermore, this technique does not allow for the automatic storage of multiple measurements and precise points of interest selected at different times, requiring the patient to be physically present at every measurement [4]. Moreover, the use of analog tools like calipers has inherent limitations, such as difficulties in measuring non-linear parameters like areas or volumes and inter- and intra-operator measurement biases. Drawbacks are linked to equipment costs and challenges in capturing deeper tissues and rendering [5,6,7].

Commercially available digital systems overcome the limitations of analog techniques by not only easing the linear measurements but also enabling complex angular, surface, and volume analysis [8,9,10,11]. Up to date protocols employed to document facial and oral conditions and measure facial proportions involve the use of both intraoral and facial photographs, as well as plaster models created from dental arch impressions [12,13]. However, conventional two-dimensional (2D) photogrammetric facial images exhibit various limitations and can be prone to significant errors due to lighting deviations, parallax, and focal length distances [14]. The incorporation of scanning devices in medical research has coincided with the advancement of Computer-Aided Design and Manufacturing (CAD/CAM) technology in dentistry, providing numerous advantages for professionals. Intra/extraoral scanners and CAD/CAM systems simplify treatment planning, enhance communication with laboratories, reduce operative times, storage requirements, and treatment durations [15,16,17,18,19].

Diverse digital technologies are now available on the market for capturing 3D facial data, each operating on distinct principles to generate accurate surface representations such as laser surface scanning, multi-image photogrammetry, stereophotogrammetry, and 3D facial photography techniques [20,21,22]. Structured light scanners project specific light patterns, like grids or parallel lines, onto the patient’s face. A camera then captures the deformation of these patterns, and proprietary software calculates the 3D coordinates based on the detected distortions, making them highly accurate for intricate details in static facial analysis. Multi-image photogrammetry and stereophotogrammetry involve simultaneous capture of multiple 2D images from various angles using an array of synchronized cameras, with software algorithms reconstructing a 3D point cloud and textured surface model through triangulation and feature matching. Laser surface scanning utilizes a laser beam to systematically scan the facial surface, measuring the distance to each point to create a 3D dataset. Literature shows that these systems provide rapid execution, straightforward storage, and high accuracy and reliability [23,24,25].

Beyond surface capture, advancements include integration with Cone Beam Computed Tomography (CBCT) machines, allowing for the fusion of hard tissue volumetric data with soft tissue surface scans, creating a comprehensive virtual patient model for advanced treatment planning.

The Planmeca ProFace extraoral scanner (Planmeca USA, Inc., Hoffman Estates, IL, USA) captures a 3D image within seconds by rotating around the patient’s head. The system utilizes lasers for facial geometry scanning and digital cameras for capturing texture and color. The device’s reported spatial accuracy is 0.03 mm, as indicated by the manufacturer. The 3D photographs are processed using Planmeca Romexis software v.6.2 (Planmeca USA, Inc., Hoffman Estates, IL, USA), enabling detailed operations [26]. However, the precision of these novel tools remains to be fully established. This study aims to evaluate the accuracy of the Planmeca ProFace scanner’s digital measurements by comparing them to analog methods for both small (≤6.5 cm) and large (>6.5 cm) facial distances. Specifically, the objectives were to quantify the mean differences between the two measurement systems, statistically assess these differences, and evaluate the internal consistency and reliability of both methods for these distinct facial dimensions.

## 2. Materials and Methods

This cross-sectional comparative study was conducted at the Department of Biomedical and Dental Sciences and Morphofunctional Imaging, Messina University, Messina, Italy. Participant recruitment and data collection were carried out between January and April 2023. Participants were enrolled via a convenience sampling method, based on their availability and willingness to participate during the study period. Participants were included if they had no history of craniofacial trauma and/or congenital deformities. Individuals with facial piercings, extensive facial hair that could obscure landmarks, or any medical condition precluding safe participation were excluded. The sample size was determined based on similar previous studies. A formal a priori power calculation was not performed due to the comparative cross-sectional nature of this exploratory study. The study adhered to ethical guidelines in line with the Helsinki declaration and was approved by the ethical committee. (prot. n.95-23, 14 December 2022, Messina Local ethical committee. A.O.U. G. Martino, A.O. Papardo, A.S.P.). This manuscript was prepared in accordance with the Strengthening the Reporting of Observational Studies in Epidemiology (STROBE) guidelines. Prior to any procedures, each participant was provided with a brief written description of the study’s objective. Written informed consent was obtained from all subjects before their participation.

**Figure 1:**
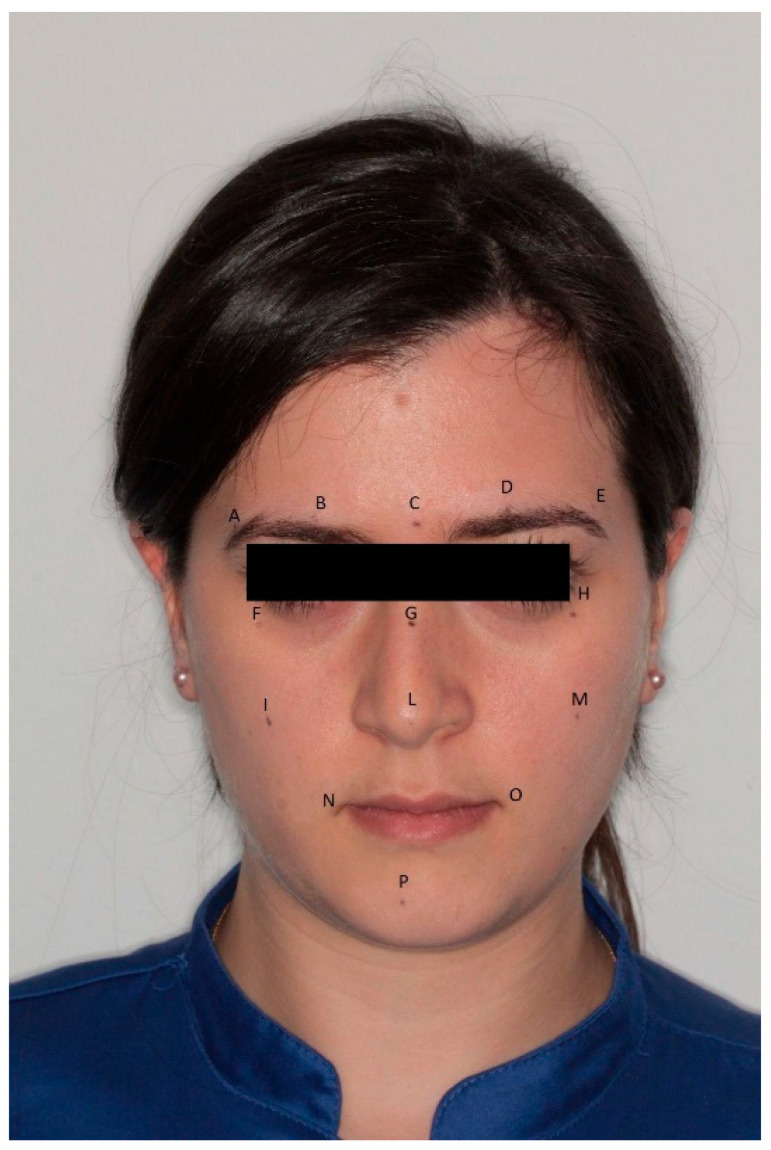
Template of the points marked on the patients’ face.

To ensure consistency and reproducibility, fourteen specific anatomical landmarks (labeled A through P) were identified and traced on each patient’s face using a fine-tipped, skin-safe marker. All marking procedures were performed by a single experienced operator and independently verified by a second, equally experienced operator to ensure accurate anatomical localization; blinding was maintained where possible, as the primary operator was unaware of the digital measurements at the time of analog measurement, and all procedures were meticulously supervised to reduce potential measurement biases (Figure 1, Table S1).

**Figure 2:**
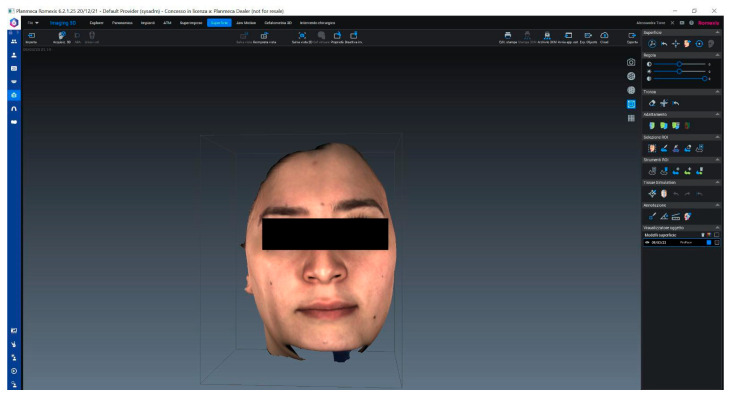
Face scan rendered through the ProFace software v.6.2.

The Planmeca ProFace extraoral scanner (Planmeca USA, Inc., Hoffman Estates, IL, USA) was employed for digital data acquisition. The scanning process involved the patient being seated in a standardized upright position, maintaining a neutral head posture, to minimize positional variations between scans. The Planmeca ProMax 3D unit, integrated with the ProFace scanner, rotated around the patient’s head, utilizing two lights, a laser, two digital cameras, and two light-emitting diodes to capture both the precise facial geometry and surface texture with color. Prior to each scanning session, the scanner was calibrated according to the manufacturer’s instructions to ensure optimal performance and adherence to its reported spatial accuracy of 0.03 mm. Scans were immediately reviewed for quality; any images exhibiting poor quality due to involuntary subject movements or artifacts were promptly retaken to ensure data integrity. The raw 3D scan data was then rendered and processed using the Planmeca Romexis software (Romexis 6.4.4.7, Planmeca USA, Inc., Hoffman Estates, IL, USA) (Figure 2).

Following digital scanning, direct analog measurements were obtained using a calibrated digital caliper (Mitutoyo 500-195-20, Mitutoyo Corporation, Kanagawa, Japan). For each of the 24 predefined distances between the marked points, three consecutive caliper measurements were taken by the same operator who performed the marking, with a brief pause between each measurement to mitigate potential human error and operator fatigue. The average of these three readings was recorded for subsequent comparison.

To address potential sources of bias, the operator was unaware of the digital measurements at the time of analog measurement. Concurrently, the same 24 linear distances were measured digitally within the Romexis Viewer software (Romexis 6.4.4.7) on the captured 3D facial models. Within the software, a dedicated linear measurement tool was utilized, and the operator carefully clicked on the precisely identified digital landmarks corresponding to the marked points on the physical face. This allowed for direct, precise measurement of the distances in the virtual 3D space. Both analog and digital measurement procedures were meticulously supervised by a second independent operator to further reduce potential measurement biases. To investigate potential differences in accuracy based on measurement length, the predefined distances were classified into two groups:

Group A (n = 12): small distances (less than or equal to 6.5 cm): AB, AC, AF, AI, CG, CL, EH, EM, IL, LM, IG, and NP.

Group B (n = 12): large distances (greater than 6.5 cm): AD, AE, AN, AP, EO, EP, GM, IP, MP, LP, AL, and EL.

This classification was established to assess the scanner’s performance across a range of facial dimensions, specifically exploring if its precision varied for smaller, more localized features versus broader facial contours.

Statistical analyses were performed using SPSS for Window (version 22) package. Descriptive statistics, including means and standard deviations, were used to characterize the study participants and the measurements obtained. Paired *t*-tests were used to assess statistically significant differences between the mean measurements obtained by the digital scanner and the analog caliper for both Group A (measurements ≤ 6.5 cm) and Group B (measurements > 6.5 cm). Data were visually inspected for outliers. Cronbach’s Alpha was calculated to assess the internal consistency of the items, for each group of measurements. Boxplots were realized to better visualize the differences between two means of measurement, in both groups. Furthermore, Bland–Altman analysis was performed to assess the agreement between the digital and analog measurement methods and to identify any systematic or proportional bias. Separate Bland–Altman plots were generated for Group A (small distances) and Group B (large distances), plotting the difference between the two measurements against their average.

A 95% confidence level was set with a 5% alpha error; statistical significance was set at *p*  <  0.05. No formal statistical adjustment for confounding variables was performed, as the primary objective was direct method comparison. The potential influence of confounders on measurements is acknowledged as a limitation in the discussion.

## 3. Results

The study included seven participants, comprising four women and three men. The age of the participants ranged from 20 to 28 years. The mean difference between the caliper and software measurements was 0.24 ± 0.9 SD (min 0.16 max 2.92) in group A and 0.71 ± 2.8 SD (min 0.02 max 4.17) in group B. Paired T tests showed a statistically significant difference between the two methods of measurements in group A (*p* = 0.016). High statistical significance was obtained in group B (*p* = 0.001) (Table 1).

Measurement results are shown in Table 2.

**Figure 3:**
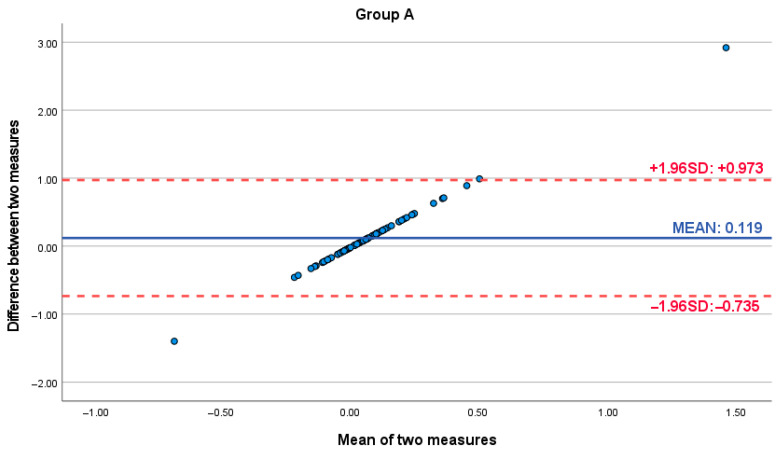
Bland–Altman plot assessing the agreement between digital and analog facial measurements for Group A (measurements ≤ 6.5 cm).

**Figure 4:**
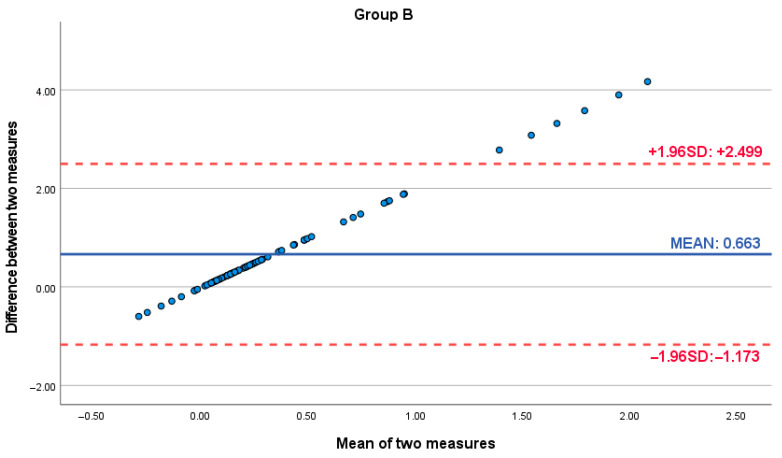
Bland–Altman plot assessing the agreement between digital and analog facial measurements for Group B (measurements > 6.5 cm).

Bland–Altman analysis was conducted to further assess the agreement and potential bias between the digital scanner and analog caliper measurements. For Group A (small distances), the Bland–Altman plot revealed a mean difference of 0.119 (95% Limits of Agreement: −0.735 to 0.973). For Group B (large distances), the mean difference was 0.663 (95% Limits of Agreement: −1.173 to 2.499). Crucially, both plots exhibited a consistent positive slope, indicating a proportional bias. This trend demonstrated that the difference between the digital and analog measurements systematically increased as the magnitude of the facial dimension being measured increased, across both small and large measurement ranges. This proportional bias, while present in Group A, became clinically significant and more pronounced for the larger measurements in Group B. Notably, within Group B, measurements exhibiting the largest discrepancies between methods were observed at the higher end of the measurement scale, falling outside the 95% Limits of Agreement and representing the furthest values from the mean difference line (Figure 3 and Figure 4).

**Figure 5:**
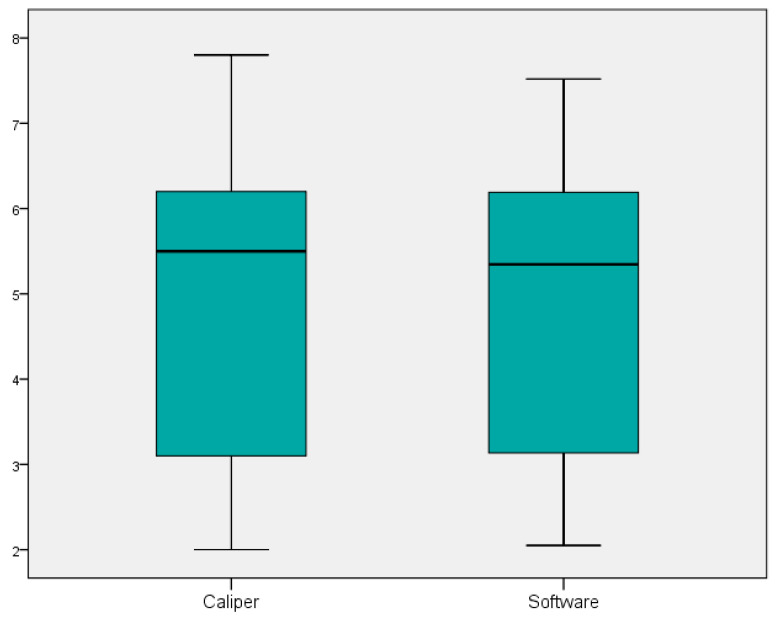
Boxplot representing the distribution and variance of the measurement differences between the digital (Planmeca ProFace) and analog (caliper) methods for Group A measurement.

**Figure 6:**
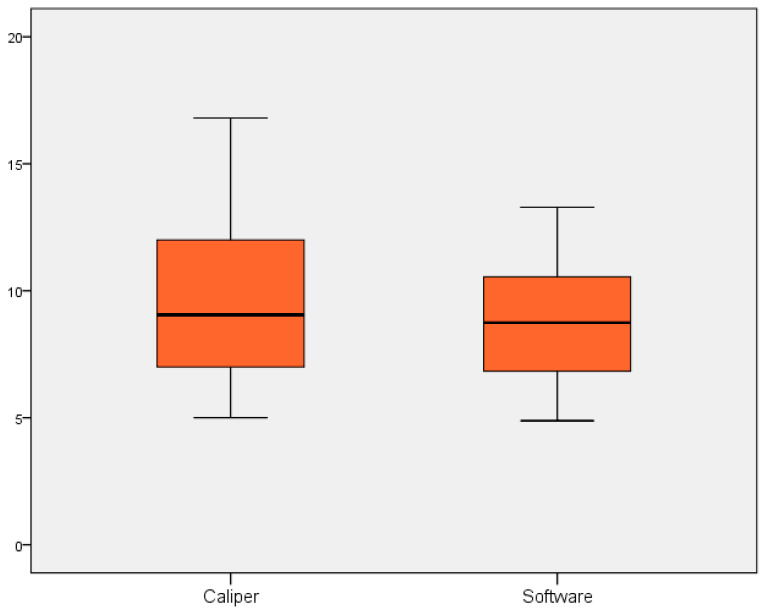
Boxplot representing the distribution and variance of the measurement differences between the digital (Planmeca ProFace) and analog (caliper) methods for Group B measurement.

The internal consistency and reliability of the measurements were assessed using Cronbach’s Alpha. For Group A (measurements ≤ 6.5 cm), Cronbach’s Alpha was calculated as (α = 0.982), indicating excellent reliability. For Group B (measurements > 6.5 cm), Cronbach’s Alpha was (α = 0.270), demonstrating low reliability. The sample distribution is listed in Figure 5 and Figure 6.

## 4. Discussion

### 4.1. Clinical Implications

Analyzing the face through 3D images can provide clinicians valuable information during treatment planning and outcomes evaluation of the therapy performed. Using a digital scanner for model acquisition offers numerous advantages compared to traditional analog techniques, requiring less time to obtain the models, being less uncomfortable for patients, facilitating communication with both patients and laboratories, and simplifying procedures [27,28,29,30]. Furthermore, most software allows the superimposition of two scans from the same patient, producing a comprehensive analysis of the differences regarding the shifts in maxillofacial components, growth trends, or treatment effects.

The application of this technology is discussed thoroughly in the literature regarding orthognathic surgery, stressing the usefulness of facial scanners in soft tissues movement prediction and programming minimal or complex bimaxillary surgeries [31]. The peri-nasal area is often affected by such surgeries and while some corrections may be employed, the literature shows how the differences may be measured and in part predicted by the means of facial scanning [32,33,34]. This cost-effective method has been successfully described in the literature for facial palsy treatment, breathing patterns morphology of the face cleft patient analysis and more [35,36,37]. Regarding aesthetic surgery, Bertazzo and D’Ornellas proposed a new protocol for rhinoseptoplasty, concluding that is reproducible and feasible in clinical practice, being a potential tool for surgical planning and comparison of results [38].

Interventions in the lower third of the face or the oral cavity can benefit from this technology as well, as described by Lin et al. The authors used this technology to create a virtual patient to restore maxillary central incisors using ceramic veneers [39]. If open or closed mouth scans are performed, scan data can also be used in surgical interventions programming allowing some sort of dynamic assessment of the intervention [40]. Moreover, due to the connection between the volume of hard and soft tissues, face scans can be a mean to assess indirectly such connection, such as the support of the dental arches through perioral tissues analysis [41].

The findings of this study, indicating statistically significant deviations between digital scanner measurements and analog caliper measurements, particularly for distances exceeding 6.5 cm, carry important clinical implications. The presence of a consistent proportional bias, as highlighted by the Bland–Altman plots, means that clinicians cannot rely on a single, fixed correction factor for digital measurements; rather, the agreement with analog methods is size-dependent. While the bias might be negligible for smaller facial features, its increasing magnitude for larger dimensions, particularly evident in Group B where the largest discrepancies occurred, necessitates heightened caution. While digital scanning offers considerable advantages in terms of efficiency and patient comfort, clinicians must be cognizant of its limitations, especially when precision is paramount for larger facial dimensions (Table 3).

For clinical procedures where even minor discrepancies can affect outcomes, such as complex orthognathic surgeries involving substantial maxillary or mandibular movements, or extensive aesthetic procedures requiring precise proportionality over larger facial regions, relying solely on digital measurements without accounting for potential inaccuracies could lead to suboptimal diagnostic interpretations or compromised therapeutic plans. To provide clearer clinical guidance, it is essential to establish a specific clinical threshold of acceptable error margins for different procedures. In highly precise interventions such as orthognathic surgery or intricate implant placements, sub-millimeter accuracy (e.g., an acceptable error margin of less than 0.5 mm) is often considered critical for optimal functional and aesthetic outcomes. In contrast, for diagnostic purposes or less critical aesthetic evaluations, a slightly larger error margin (e.g., 1–2 mm) might be tolerable. The mean difference of 0.71 ± 2.8 SD (with a maximum deviation of 4.17 mm) observed for Group B measurements suggests that these deviations may exceed the acceptable clinical thresholds for procedures demanding high precision.

This is also true in clinical scenarios such as full-mouth rehabilitations and full-arch fixed restorations on implants. In these complex treatments, precise assessment and planning of facial modifications and changes in the vertical dimension of occlusion (VDO) are paramount. Digital systems excel in providing comprehensive 3D data, enabling advanced angular, surface, and volume analysis, which surpasses the capabilities of traditional analog techniques for planning, anticipating soft tissue responses and overall facial aesthetic changes associated with VDO adjustments. While digital models facilitate enhanced patient communication, crucial for discussing significant facial and occlusal changes, clinicians must be mindful of the observed limitations.

### 4.2. Technical Issues

While the technical advancements in bone segmentation and treatment simulation are eased by the rigid nature of the bone tissue, soft tissue simulation may be compromised by the multiple interactions between skin layers, muscles, fascia, and bone and may still vary due to its dynamic nature. Despite the results, prediction is still seen as an approximation and will still have to rely on the refinement of the algorithms; the superimposition of pre-intervention and post-intervention scans may give the researchers and clinicians valuable information, thus it is imperative for these newly introduced commercial tools to be reliable and provide accurate imaging to be used in daily practice [31].

The reliability of 3D scanner acquisition has been analyzed in the literature [42,43,44,45]. Stereophotogrammetry has proved to be precise, with random errors of less than 1 mm, while laser scanners were found to be affected by a mean scanning error of 1.1 mm [42,43,44,45]. The precision of the Planmeca ProFace system employed in this research has never been compared in the literature to analog instruments.

D. K. Liberton et al. assessed the precision difference between three different scanners: the 3dMDface stereophotogrammetry system (3dMD, Atlanta, GA, USA), the Vectra H1 handheld photogrammetry system (Canfield Scientific, Parsippany, NJ, USA), and the ProFace laser scanning system. The results showed high accuracy for every system analyzed, although the 3dMD and Vectra results showed higher result concordance [46]. Nonetheless, it should be noted that the software version used to render the results was not specified. While the hardware may not change, software algorithms are frequently updated and may vary from one version to another, possibly affecting the results or future comparisons.

Another research has analyzed the measurement differences obtained with Planmeca ProFace system compared with different types of scanners, including EinScan Pro (Shining 3D Tech. Co., Ltd., Hangzhou, China), EinScan Pro 2X Plus (Shining 3D Tech. Co., Ltd., Hangzhou, China), iPhone X (Apple Store, Cupertino, CA, USA). Variances in scan precision were recorded, with the Planmeca ProMax scanner being the least accurate among them [29]. The authors suggest that these disparities can be attributed to the distinct technologies employed by these instruments [29].

The results of our research show statistically significant differences between face scan measurements and analog caliper measurements, both for length > 6.5 cm and ≤6.5 cm. These results seem to be in accordance with literature reports [29]. The differences analyzed are stronger in group B (*p* < 0.001), than in group A (*p* < 0.05).

Cronbach’s alpha showed how the instruments results tend to be similar in Group A (0.982) rather than group B (0.270), underlining how, for measurements exceeding 6.5 cm, the scanner’s precision is lower. This is further confirmed by the mean difference between the two methods in group A (0.23 cm) and group B (1.38 cm). Nonetheless, the data taken into consideration are mean values: the potential inaccuracy of the Planmeca scanner could be attributed to scanning from a short distance, resulting in a narrower field of vision [5,6,7,43].

Despite these shortcomings, analog hand measurements have higher inter- and intra- operator variability when compared to a system that scans the patient always in the same position. While the points marked by software manually may always represent a bias, this seems to be lower than caliper measurements and possess the advantage to repeat the marking and measurements without the physical presence of the patient.

It is important to acknowledge that the accuracy of 3D models and measurements derived from digital scanning systems can be influenced by version updates of the associated software. Subsequent software iterations may incorporate refined algorithms for image processing, 3D reconstruction, landmark identification, or measurement calculations. These improvements, while generally beneficial, could potentially alter the precision and reproducibility observed in the current study. Future research or clinical applications should therefore consider the specific software version used and its potential impact on reported accuracy metrics, ensuring consistency or accounting for variations introduced by updates.

### 4.3. Future Applications and Limitations

As such, we strongly advise physicians regularly utilizing the Planmeca ProFace scanner to incorporate the observed mean error into their evaluation of facial proportions and symmetry, particularly for measurements greater than 6.5 cm. This might necessitate a combined approach, where critical large measurements are either validated with analog tools or integrated into a workflow that accounts for the scanner’s known variability in those ranges.

The dynamic and complex nature of soft tissues, influenced by multiple interactions between skin layers, muscles, fascia, and underlying bone, remains a challenge for precise digital simulation. While current algorithms offer approximations, future research is imperative to refine these computational models, leading to more accurate and predictable soft tissue behavior in virtual planning environments. This includes developing algorithms that better account for tissue elasticity, gravity, and patient-specific variations, which are critical for predicting post-treatment outcomes in orthognathic and aesthetic surgeries.

Furthermore, facial scanning technologies are increasingly being integrated into augmented reality (AR) frameworks, holding immense promise for enhancing the precision of dental and maxillofacial treatments [47]. AR overlays virtual plans onto the real patient, providing real-time guidance during procedures. However, the observed discrepancies in larger measurements from systems like the Planmeca ProFace suggest that further validation and potential calibration adjustments are crucial before this technology can be universally recommended for highly precise real-time registration and alignment of virtual and real images in AR-guided surgeries. Future research should focus on developing robust registration algorithms that can compensate for scanner-specific inaccuracies, thereby maximizing the potential of AR in clinical settings.

The primary limitation of this study is its relatively small sample size, which naturally restricts the generalizability of our findings to broader clinical populations. Future investigations should therefore prioritize larger, multicenter clinical trials to validate these outcomes across a more diverse demographic range, potentially including patients with varying craniofacial morphologies, ages, and pathologies. Additionally, while our study meticulously controlled for operator bias by utilizing a single, supervised operator for all measurements, future research could explore inter-operator and intra-operator variability for both digital and analog methods across multiple clinical sites to provide a more comprehensive understanding of measurement consistency, considering the variability in manual landmark identification, operator fatigue, and/or scanner-specific artifacts. Additionally, the use of a convenience sampling method, which may introduce selection bias, is a limitation that affects the generalizability of the findings to a broader population.

A point for future investigation is the identification of a consistent proportional bias in the Bland–Altman analysis. This positive slope, indicating increasing discrepancies with larger measurements, suggests an intrinsic characteristic of the digital measurement process compared to analog methods. Future research should aim to investigate the underlying causes of this proportional bias within 3D scanning technology and explore the development of size-specific correction algorithms or calibration methods to enhance the overall accuracy and interchangeability of digital measurements across the entire range of facial dimensions. Comparative studies involving a wider array of commercially available 3D facial scanners, such as comparisons with high-precision structured light scanners or advanced stereophotogrammetry systems, would also be beneficial to establish a broader benchmark for accuracy and precision in different measurement ranges.

Finally, research into the development of standardized clinical protocols for 3D facial scanning, including patient positioning, scan acquisition parameters, and post-processing techniques, is warranted to minimize external variables and enhance the reproducibility and reliability of digital facial measurements in routine clinical practice.

## 5. Conclusions

This study evaluated the Planmeca ProFace 3D digital facial scanner against analog caliper measurements, revealing a critical dependency of accuracy on measurement length. While the digital scanner demonstrated high reliability, significant discrepancies were observed for facial distances exceeding 6.5 cm. Conversely, for measurements ≤ 6.5 cm, digital and analog methods showed strong agreement. These findings highlight that while digital scanning offers considerable clinical advantages, its application for precise large facial measurements requires careful consideration. To ensure accuracy, particularly for these critical large facial dimensions, clinicians may need to combine digital measurements with analog validation or apply specific compensatory strategies.

## Figures and Tables

**Table 1:** Confidence intervals for mean differences and paired *t*-test results.

		Differences in Pairs—Group A					Paired *t*-Test	*p*-Value
		N	Mean Difference	SD	95% Confidence Interval			
					Lower	Upper		
Pair	Caliper-Software Romexis v.62	12	0.24	0.9	0.016	0.464	2.335	0.016
		Differences in Pairs—Group B					Paired *t*-Test	*p*-Value
		N	Mean Difference	SD	95% Confidence Interval			
					Lower	Upper		
Pair	Caliper-Software	12	0.71	2.8	0.248	1.172	3.688	0.001

**Table 2:** Measurement results.

Measurement Name	Caliper Mean	Caliper Std	Caliper Min	Caliper Max	Digital Mean	Digital Std	Digital Min	Digital Max
AB	3.39	0.43	2.40	4.00	3.26	0.34	2.40	3.41
AC	7.21	1.14	6.00	8.20	6.26	0.50	6.00	7.31
AD	11.07	1.41	9.00	13.00	9.42	1.27	8.45	11.11
AE	14.04	1.48	12.00	16.20	11.05	1.07	10.30	12.30
AF	2.54	0.59	2.00	3.50	2.57	0.48	2.05	3.31
AI	6.24	0.19	6.00	6.50	6.26	0.27	6.18	6.54
AL	9.29	1.07	8.40	10.00	9.37	1.12	8.14	10.17
AN	9.14	0.38	8.50	10.00	9.25	0.23	9.05	9.28
AP	13.33	1.38	12.50	15.40	12.34	0.37	12.22	13.32
CG	3.23	0.34	3.20	4.00	3.17	0.38	3.05	4.08
CL	6.37	0.49	5.30	6.50	5.47	1.35	3.58	6.46
EH	3.01	0.32	2.20	3.00	3.05	0.34	2.27	3.24
EL	9.21	1.00	8.20	10.30	9.28	1.03	8.34	10.20
EM	6.07	0.42	5.50	6.50	6.13	0.34	5.53	6.49
EO	9.11	1.00	8.00	10.00	9.24	0.28	8.39	9.27
EP	13.27	2.02	12.00	17.20	12.37	0.53	12.13	13.29
GM	6.06	1.09	5.00	7.00	6.36	1.16	5.28	7.08
IG	6.00	1.08	5.20	7.00	6.14	1.00	5.09	7.50
IL	6.20	1.28	5.00	7.50	6.16	1.34	4.35	7.52
IP	7.03	1.03	6.40	8.30	7.09	0.57	6.06	8.28
LM	6.26	1.37	4.50	7.50	6.18	1.28	4.56	7.08
LP	6.56	0.23	6.30	7.00	6.26	0.40	6.05	7.27
MP	7.36	1.08	6.40	8.20	7.08	1.02	6.36	7.46
NP	4.54	0.49	4.00	5.50	4.31	0.48	4.32	5.32

**Table 3:** Advantages, disadvantages, indications, and limitations of digital vs. analog measuring methods.

	Digital Measuring Methods (e.g., Planmeca ProFace Scanner)	Analog Measuring Methods (e.g., Caliper)
Advantages	-Efficiency: Faster data acquisition. -Comfort: Non-invasive, increased patient comfort. -Detail: Captures comprehensive 3D facial data, including texture and color. -Analysis: Enables complex angular, surface, and volume analysis. -Communication: Facilitates better patient communication.	-Precision: High accuracy for small, linear measurements when performed meticulously. -Simplicity: No specialized software or advanced training required beyond basic instrument use. -Cost-Effective: Generally lower initial equipment cost.
Disadvantages	-Accuracy Variability: Can show significant deviations for larger measurements (6.5 cm). -Soft Tissue Challenges: Simulation can be complex due to the dynamic nature of soft tissues. -Cost: Higher initial investment in equipment and software. -Learning Curve: Requires specific training for operation and software interpretation.	-Time-Consuming: Slower data acquisition. -Operator Dependent: Highly susceptible to inter-operator variability and human error. -Limited Scope: Primarily provides linear measurements, lacks 3D data for complex analysis. -Patient Discomfort: Can be invasive for certain measurements.
Indications	Comprehensive Facial Analysis: Ideal for detailed pre-and post-treatment evaluation in orthodontics, orthognathic surgery, and aesthetic procedures. -Treatment Planning: For complex cases requiring 3D visualization and simulation, especially for minor changes. -Patient Education: Enhances understanding of planned interventions.	-Validation: For verifying critical linear measurements, especially those where digital methods may show less precision (e.g., larger distances). -Basic Linear Measurements: For simple, direct measurements where 3D data is not critical. -Resource-Limited Settings: Where digital equipment is not available or feasible.
Limitations	-Precision for Large Distances: May lack sufficient precision for measurements exceeding 6.5 cm, requiring compensatory strategies. -Soft Tissue Dynamics: Challenges in accurately predicting dynamic soft tissue changes.	Inability to Capture 3D: Does not provide volumetric or surface data. -Limited Accessibility: Some facial areas may be difficult to access for direct measurement. -Replication: Difficult to replicate exact measurement points consistently across multiple sessions.

## Supplementary Materials

The following supporting information can be downloaded at: https://www.mdpi.com/article/10.3390/dj13090395/s1, Table S1: Facial landmarks used for measurements.

## References

[1] Gahl W.A., Mulvihill J.J., Toro C., Markello T.C., Wise A.L., Ramoni R.B., Adams D.R., Tifft C.J. (2016). The NIH Undiagnosed Diseases Program and Network: Applications to modern medicine. Mol. Genet. Metab..

[2] Kesterke M.J., Raffensperger Z.D., Heike C.L., Cunningham M.L., Hecht J.T., Kau C.H., Nidey N.L., Moreno L.M., Wehby G.L., Marazita M.L. (2016). Using the 3D Facial Norms Database to investigate craniofacial sexual dimorphism in healthy children, adolescents, and adults. Biol. Sex Differ..

[3] Claes P., Liberton D.K., Daniels K., Rosana K.M., Quillen E.E., Pearson L.N., McEvoy B., Bauchet M., Zaidi A.A., Yao W. (2014). Modeling 3D facial shape from DNA. PLoS Genet.

[4] Sforza C., Ferrario V.F. (2006). Soft-tissue facial anthropometry in three dimensions: From anatomical landmarks to digital morphology in research, clinics and forensic anthropology. J. Anthropol. Sci..

[5] Jones T., Leary S., Atack N., Chawla O., Ness A., Ireland T., Sandy J. (2016). Are photographs a suitable alternative to dental study casts when assessing primary surgical outcome in children born with unilateral cleft lip and palate?. Eur. J. Orthod..

[6] Nollet P.J., Katsaros C., van ‘t Hof M.A., Bongaarts C.A., Semb G., Shaw W.C., Kuijpers-Jagtman A.M. (2004). Photographs of study casts: An alternative medium for rating dental arch relationships in unilateral cleft lip and palate. Cleft. Palate Craniofac. J..

[7] Baheti M.J., Soni U.N., Gharat N.V., Mahagaonkar P., Khokhani R., Dash S. (2015). Intra-oral Scanners: A New Eye in Dentistry. Austin. J. Orthopade Rheumatol..

[8] Weinberg S.M., Naidoo S., Govier D.P., Martin R.A., Kane A.A., Marazita M.L. (2006). Anthropometric precision and accuracy of digital three-dimensional photogrammetry: Comparing the Genex and 3dMD imaging systems with one another and with direct anthropometry. J. Craniofac. Surg..

[9] Hong C., Choi K., Kachroo Y., Kwon T., Nguyen A., McComb R., Moon W. (2017). Evaluation of the 3dMDface system as a tool for soft tissue analysis. Orthod. Craniofac. Res..

[10] Gibelli D., Codari M., Rosati R., Dolci C., Tartaglia G.M., Cattaneo C., Sforza C. (2015). A Quantitative Analysis of Lip Aesthetics: The Influence of Gender and Aging. Aesthetic. Plast. Surg..

[11] Codari M., Pucciarelli V., Pisoni L., Sforza C. (2015). Laser scanner compared with stereophotogrammetry for measurements of area on nasal plaster casts. Br. J. Oral. Maxillofac. Surg..

[12] Zimmermann M., Mehl A., Mormann W.H., Reich S. (2015). Intraoral scanning systems—A current overview. Int. J. Comput. Dent..

[13] Alghazzawi T.F. (2016). Advancements in CAD/CAM technology: Options for practical implementation. J. Prosthodont. Res..

[14] Ambrosio E.C.P., Fusco N.D.S., Carrara C.F.C., Bergamo M.T., Neto N.L., Cruvinel T., Rios D., Almeida A., Soares S., Machado M. (2022). Digital Volumetric Monitoring of Palate Growth in Children With Cleft Lip and Palate. J. Craniofac. Surg..

[15] Rando G.M., Ambrosio E.C.P., Jorge P.K., Prado D.Z.A., Falzoni M.M.M., Carrara C.F.C., Soares S., Machado M., Oliveira T.M. (2018). Anthropometric Analysis of the Dental Arches of Five-Year-Old Children With Cleft Lip and Palate. J. Craniofac. Surg..

[16] Gomez-Polo M., Gomez-Polo C., Del Rio J., Ortega R. (2018). Stereophotogrammetric impression making for polyoxymethylene, milled immediate partial fixed dental prostheses. J. Prosthet. Dent..

[17] De Menezes M., Ceron-Zapata A.M., Lopez-Palacio A.M., Mapelli A., Pisoni L., Sforza C. (2016). Evaluation of a Three-Dimensional Stereophotogrammetric Method to Identify and Measure the Palatal Surface Area in Children With Unilateral Cleft Lip and Palate. Cleft. Palate Craniofac. J..

[18] Penarrocha-Diago M., Balaguer-Marti J.C., Penarrocha-Oltra D., Balaguer-Martinez J.F., Penarrocha-Diago M., Agustin-Panadero R. (2017). A combined digital and stereophotogrammetric technique for rehabilitation with immediate loading of complete-arch, implant-supported prostheses: A randomized controlled pilot clinical trial. J. Prosthet. Dent..

[19] Deli R., Gioia E.D., Galantucci L.M., Percoco G. (2011). Accurate facial morphologic measurements using a 3-camera photogrammetric method. J. Craniofac. Surg..

[20] Plooij J.M., Maal T.J., Haers P., Borstlap W.A., Kuijpers-Jagtman A.M., Berge S.J. (2011). Digital three-dimensional image fusion processes for planning and evaluating orthodontics and orthognathic surgery. A systematic review. Int. J. Oral. Maxillofac. Surg..

[21] Gwilliam J.R., Cunningham S.J., Hutton T. (2006). Reproducibility of soft tissue landmarks on three-dimensional facial scans. Eur. J. Orthod..

[22] Menendez Lopez-Mateos M.L., Carreno-Carreno J., Palma J.C., Alarcon J.A., Menendez Lopez-Mateos C., Menendez-Nunez M. (2019). Three-dimensional photographic analysis of the face in European adults from southern Spain with normal occlusion: Reference anthropometric measurements. BMC Oral Health.

[23] Coachman C., Georg R., Bohner L., Rigo L.C., Sesma N. (2020). Chairside 3D digital design and trial restoration workflow. J. Prosthet. Dent..

[24] Richert R., Goujat A., Venet L., Viguie G., Viennot S., Robinson P., Farges J.C., Fages M., Ducret M. (2017). Intraoral Scanner Technologies: A Review to Make a Successful Impression. J. Healthc. Eng..

[25] Goracci C., Franchi L., Vichi A., Ferrari M. (2016). Accuracy, reliability, and efficiency of intraoral scanners for full-arch impressions: A systematic review of the clinical evidence. Eur. J. Orthod..

[26] Lawson N.C., Burgess J.O. (2015). Clinicians reaping benefits of new concepts in impressioning. Compend. Contin. Educ. Dent..

[27] Aragon M.L., Pontes L.F., Bichara L.M., Flores-Mir C., Normando D. (2016). Validity and reliability of intraoral scanners compared to conventional gypsum models measurements: A systematic review. Eur. J. Orthod..

[28] Lo Giudice R., Lizio A., Cervino G., Fabiana N., Francesco P., Ausiello P., Cicciu M. (2018). The Horizontal Root Fractures. Diagnosis, Clinical Management and Three-Year Follow-Up. Open Dent. J..

[29] Lo Giudice R., Galletti C., Tribst J.P.M., Melenchón L.P., Matarese M., Miniello A., Cucinotta F., Salmeri F. (2022). In Vivo Analysis of Intraoral Scanner Precision Using Open-Source 3D Software. Prosthesis.

[30] Amornvit P., Sanohkan S. (2019). The Accuracy of Digital Face Scans Obtained from 3D Scanners: An In Vitro Study. Int. J. Environ. Res. Public Health.

[31] Awad D., Reinert S., Kluba S. (2022). Accuracy of Three-Dimensional Soft-Tissue Prediction Considering the Facial Aesthetic Units Using a Virtual Planning System in Orthognathic Surgery. J. Pers. Med..

[32] Sabri H., Tehranchi A., Sarkarat F. (2024). 3-dimensional analysis of nasal soft tissue alterations following maxillary Lefort I advancement with and without impaction using 3D photogrammetry scanner. Oral. Maxillofac. Surg..

[33] Rauso R., Tartaro G., Nicoletti G.F., Fragola R., Lo Giudice G., Santagata M. (2022). Alar cinch sutures in orthognathic surgery: Scoping review and proposal of a classification. Int. J. Oral. Maxillofac. Surg..

[34] Krijt L.L., Kapetanovic A., Sijmons W.J.L., Bruggink R., Baan F., Berge S.J., Noverraz R.R.M., Xi T., Schols J. (2023). What is the impact of miniscrew-assisted rapid palatal expansion on the midfacial soft tissues? A prospective three-dimensional stereophotogrammetry study. Clin. Oral. Investig..

[35] Sforza C., Ulaj E., Gibelli D.M., Allevi F., Pucciarelli V., Tarabbia F., Ciprandi D., Dell’Aversana Orabona G., Dolci C., Biglioli F. (2018). Three-dimensional superimposition for patients with facial palsy: An innovative method for assessing the success of facial reanimation procedures. Br. J. Oral. Maxillofac. Surg..

[36] Cheng B., Mohamed A.S., Habumugisha J., Guo Y., Zou R., Wang F. (2023). A Study of the Facial Soft Tissue Morphology in Nasal- and Mouth-Breathing Patients. Int. Dent. J..

[37] Olmos M., Matta R., Buchbender M., Jaeckel F., Nobis C.P., Weber M., Kesting M., Lutz R. (2023). 3D assessment of the nasolabial region in cleft models comparing an intraoral and a facial scanner to a validated baseline. Sci. Rep..

[38] Bertazzo T.L., D’Ornellas M.C. (2023). Protocol for capturing 3D facial meshes for rhinoseptoplasty planning. Braz. J. Otorhinolaryngol..

[39] Lin W.S., Harris B.T., Phasuk K., Llop D.R., Morton D. (2018). Integrating a facial scan, virtual smile design, and 3D virtual patient for treatment with CAD-CAM ceramic veneers: A clinical report. J. Prosthet. Dent..

[40] Zhang Y., Tao B., Wang F., Wu Y. (2023). Integrating a mouth opening assessment of virtual patients to prevent intraoperative challenges during treatment. J. Prosthet. Dent..

[41] Natale M., Soardi C.M., Saleh M.H.A., Ponzi A., Tagliaferri D., Filannino F.M., Fontana F., Decker A., Marinotti F., d’Ambrosio A. (2024). Immediate Implant Placement Using the Socket Shield Technique: Clinical, Radiographic, and Volumetric Results Using 3D Digital Techniques-A Case Series. Int. J. Periodontics Restorative Dent..

[42] Gibelli D., Pucciarelli V., Poppa P., Cummaudo M., Dolci C., Cattaneo C., Sforza C. (2018). Three-dimensional facial anatomy evaluation: Reliability of laser scanner consecutive scans procedure in comparison with stereophotogrammetry. J. Craniomaxillofac. Surg..

[43] Marmulla R., Hassfeld S., Luth T., Muhling J. (2003). Laser-scan-based navigation in cranio-maxillofacial surgery. J. Craniomaxillofac. Surg..

[44] de Menezes M., Rosati R., Ferrario V.F., Sforza C. (2010). Accuracy and reproducibility of a 3-dimensional stereophotogrammetric imaging system. J. Oral Maxillofac. Surg..

[45] Gibelli D., Pucciarelli V., Cappella A., Dolci C., Sforza C. (2018). Are Portable Stereophotogrammetric Devices Reliable in Facial Imaging? A Validation Study of VECTRA H1 Device. J. Oral Maxillofac. Surg..

[46] Liberton D.K., Mishra R., Beach M., Raznahan A., Gahl W.A., Manoli I., Lee J.S. (2019). Comparison of Three-Dimensional Surface Imaging Systems Using Landmark Analysis. J. Craniofac. Surg..

[47] Puleio F., Tosco V., Pirri R., Simeone M., Monterubbianesi R., Lo Giudice G., Lo Giudice R. (2024). Augmented Reality in Dentistry: Enhancing Precision in Clinical Procedures-A Systematic Review. Clin. Pract..

