# Supplementary material for: Precision in Facial Measurements: Comparative Analysis Between a Digital 3D Scanner and an Analog Instrument
Source: Dent J (Basel). 2025 Aug 29;13(9):395. doi: 10.3390/dj13090395 (PMC12468633; doi:10.3390/dj13090395)
Supplement: Supplementary file 1 [file dentistry-13-00395-s001.zip › dentistry-3649062-supplementary.pdf]

Landmark point	Anatomical reference
A	Outer end of the right eyebrow
B	Right supraorbital ridge
C	Glabella
D	Left supraorbital ridge
E	Outer end of the left eyebrow
F	Right lower orbital rim. Zygomaticomaxillary suture
G	Rhinion
H	Left lower orbital rim. Zygomaticomaxillary suture
I	The highest point on the right maxillary alveolar process
L	Nasal tip
M	The highest point on the left maxillary alveolar process
N	Right mouth corner
O	Left mouth corner
P	Pogonion

Supplemental Table S1: Facial landmarks used for measurements
